# Titanium Nanoparticles Enhance Production and Suppress Stabilin-1-Mediated Clearance of GDF-15 in Human Primary Macrophages

**DOI:** 10.3389/fimmu.2021.760577

**Published:** 2021-12-15

**Authors:** Lina S. Silva-Bermudez, Tatyana N. Sevastyanova, Christina Schmuttermaier, Carolina De La Torre, Leonie Schumacher, Harald Klüter, Julia Kzhyshkowska

**Affiliations:** ^1^ Institute of Transfusion Medicine and Immunology, Medical Faculty Mannheim, Heidelberg University, Mannheim, Germany; ^2^ German Red Cross Blood Service Baden-Württemberg – Hessen, Mannheim, Germany; ^3^ Microarray Analytics – NGS Core Facility (IKC), Medical Faculty Mannheim, University of Heidelberg, Mannheim, Germany

**Keywords:** titanium, nanoparticle, macrophage, endocytosis, scavenger receptor, growth factor

## Abstract

Macrophages are key innate immune cells that mediate implant acceptance or rejection. Titanium implants degrade over time inside the body, which results in the release of implant wear-off particles. Titanium nanoparticles (TiNPs) favor pro-inflammatory macrophage polarization (M1) and lower tolerogenic activation (M2). GDF-15 regulates immune tolerance and fibrosis and is endocytosed by stabilin-1. How TiNPs affect the healing activities of macrophages and their release of circulating cytokines is an open question in regenerative medicine. In this study for the first time, we identified the transcriptional program induced and suppressed by TiNPs in human pro-inflammatory and healing macrophages. Microarray analysis revealed that TiNPs altered the expression of 5098 genes in M1 (IFN-γ-stimulated) and 4380 genes in M2 (IL-4–stimulated) macrophages. 1980 genes were differentially regulated in both M1 and M2. Affymetrix analysis, confirmed by RT-PCR, demonstrated that TiNPs upregulate expression of GDF-15 and suppress stabilin-1, scavenger receptor of GDF-15. TiNPs also significantly stimulated GDF-15 protein secretion in inflammatory and healing macrophages. Flow cytometry demonstrated, that scavenging activity of stabilin-1 was significantly suppressed by TiNPs. Confocal microscopy analysis showed that TiNPs impair internalization of stabilin-1 ligand acLDL and its transport to the endocytic pathway. Our data demonstrate that TiNPs have a dual effect on the GDF-15/stabilin-1 interaction in macrophage system, by increasing the production of GDF-15 and suppressing stabilin-1-mediated clearance function. In summary, this process can result in a significant increase of GDF-15 in the extracellular space and in circulation leading to unbalanced pro-fibrotic reactions and implant complications.

## Introduction

Implantation of biomedical devices is one of the most frequently performed procedures in fields such as orthopedics and dentistry (1, 2). Macrophages play a critical role in the modulation of implant microenvironment (3). Upon implantation, monocytes are recruited from the circulation to differentiate into macrophages and react against the foreign body. The recognition of the material as a foreign body in the tissue encompasses macrophage interaction with the implant surface. This interaction favors the release of chemokines, which recruit additional macrophages and other immune cells leading to acute inflammation (4). Among the many different subtypes of macrophages, there are two activation patterns in which macrophages can be found *in vitro*, according to their function: pro-inflammatory (M1) or regulatory (M2). M1 differentiation is obtained in response to interferon-γ (INF-γ) and lipopolysaccharide and contributes to acute cytotoxic responses. M2 differentiation occurs in response to interleukin 4 (IL-4) or IL-13 and it is associated with healing responses, promoting local tissue remodeling (5). M0 are unpolarized or uncommitted macrophages that have neither pro-inflammatory nor anti-inflammatory characteristics, and which differentiation is driven by M-CSF (6). An adequate balance between these subtypes of macrophage influences the degree of osseointegration (7). In contrast, a dysregulated immune response leads to complications, such as infection and aseptic loosening, which are indications for surgical revision (8, 9). Implant revision not only negatively affects the patient’s quality of life but also aggravates the economic burden due to an increase in hospitalization rates and in the necessity of reintervention (10).

Titanium is a widely used material due to its advantageous biocompatibility properties, corrosion resistance, and low magnetic susceptibility (1). However, with time and friction, it generates wear-off particles, also known as implant debris. Particles of a diameter smaller than 1 µm, or nanoparticles, generate the most biological toxicity and can induce mutations (11, 12). Titanium nanoparticles (TiNPs) are released *in vitro*, even in the absence of implant friction (13). TiNPs are internalized by macrophages in a dose-dependent matter and promote sustained production of pro-inflammatory factors, which can result in acute and chronic inflammation (14–17). For instance, lysosomal cathepsins are released, activating nod-like receptor protein 3 (NRLP3) inflammasome, and as a consequence, IL-1β release, promoting osteoclast differentiation and peri-implant osteolysis (18). Pajarinen et al, showed an exacerbated pro-inflammatory profile in human CD14+ derived M1 and a suppressed inflammatory response in M2, in response to co-culturing with TiNPs (19). Supporting this preferential polarization, oxidative stress, mainly mediated by M1 phenotype, is a frequent response to TiNPs (17).

Despite isolated reports about the pro-inflammatory effects of TiNPs on macrophages, no systematic analysis has been performed to date to show the full transcriptional program affected by TiNPs in pro-inflammatory and healing macrophages. In this study for the first time, we addressed the question about the stimulatory and suppressive effect of TiNPs on the transcriptional program in human pro-inflammatory and healing macrophages. We found that TiNPs stimulate all subtypes of macrophages to produce growth differentiation factor 15 (GDF-15), a cytokine involved in the regulation of tissue remodeling, healing, and angiogenesis, with growing evidence about its implication in pathology. GDF-15 is also known for being a multifunctional cytokine mainly expressed and secreted during stress conditions. Although its role is still controversial, GDF-15 is hypothesized to be part of a negative feedback mechanism to counteract inflammatory reactions (20). We also found that TiNPs have a specific suppressing effect on the scavenging function of stabilin-1, which is a clearance receptor of GDF-15. This dual effect can result in the uncontrolled increase of GDF-15 levels in the tissues in close proximity to implants as well as in the circulation of patients with implants.

## Methods

### Monocyte Isolation and Generation of Macrophages

Monocytes were isolated from Buffy coats obtained from healthy blood donors out of the German Red Cross Blood Service Baden-Württemberg – Hessen after informed consent, as described previously (21). The isolation was carried out using CD14 positive selection (Miltenyi Biotec), resulting in 90–98% monocyte purity, controlled by flow cytometry. The cells were seeded into cell culture dishes in customized serum-free medium (SFM from Gibco) supplemented with 5 mM glucose at a concentration of 1x10^6^ cells/mL. Macrophage were differentiated in the presence of M-CSF at 5 ng/mL (Peprotech; #A300-25B) and Dexamethasone 10^-8^ M (Sigma, #D2915). For M1 polarization IFN-γ was used at the concentration of 100 ng/mL (Peprotech; # 300-02), for M2 polarization, IL-4 was used at the concentration of 10 ng/mL (Peprotech; #200-04). No cytokines were added for M0 differentiation.

### Stimulation With TiNPs

Titanium nanoparticles were purchased from NanoAmor Europe, France. The stock solution was initially diluted to 1:4 in DPBS, followed by another 10-fold dilution in 5 mM glucose SFM media to achieve the final dilution factor (1:4000). The TiNPs were added to a final concentration of 0,0100% (100ppm). The corresponding dilution was sterilized *via* UV.

The conditions were maintained for 6 days with 7.5% CO2 at 37°C. A daily microscopic check-up was performed to evaluate the health status of the cells. Additionally, the viability of macrophages was assessed using Alamar Blue test.

### RNA Isolation and Affymetrix Chip Analysis

After incubation with 0,0100% (100ppm) TiNPs for 6 days, cells were lysed in TRK lysis buffer and RNA was isolated using E.Z.N.A. Total RNA kit I (Omega Bio-tek, USA) according to the manufacturer’s instructions. The concentration of isolated RNA was determined with a Tecan Infinite^®^ 200. RNA was tested by capillary electrophoresis on an Agilent 2100 bioanalyzer (Agilent) and high-quality was confirmed. Hybridization of probes was done using arrays of human HuGene-1_0-st-type (Affymetrix, High Wycombe, UK). Biotinylated antisense cRNA was then prepared according to the Affymetrix standard labeling protocol with the GeneChip^®^ WT Plus Reagent Kit and the GeneChip^®^ Hybridization, Wash and Stain Kit (both from Affymetrix, Santa Clara, USA). Afterward, the hybridization on the chip was performed on a GeneChip Hybridization oven 640, then dyed in the GeneChip Fluidics Station 450 and thereafter scanned with a GeneChip Scanner 3000. All of the equipment used was from the Affymetrix-Company (Affymetrix, High Wycombe, UK). All the necessary procedures needed for hybridization and scanning of chips were performed in the Affymetrix Core Facility of Medical Research Center, Medical Faculty Mannheim.

### RT-PCR

cDNA synthesis was performed using SensiFAST cDNA Synthesis Kit from BIOLINE according to the manufacturer’s instructions. The obtained cDNA was diluted 10 times and 1 μL was used for RT-PCR. Levels of mRNA from stabilin-1 and GDF-15, were quantified using TaqMan PCR primer mix (Eurofins, Germany) in the standard conditions. Primer sequences and probes are shown from the 5′ end to 3′ end direction.

For hsSTAB-1 the following sequence was used: FP: GCGACACCTTTTGTGAAC, RP: ATGCTTCTGCTTTCAGCC, Pr: FAM TTCGATGACTCACTGCTGGAGGAGGACTT.

For 18srRNA: FP: CCATTCGAACGTCTGCCCTAT, RP: TCACCCGTGGTCACCATG, Pr: ACTTTCGATGGTAGTCGCCGTGCCT.

Ready-to-use Taqman master mixes were used for GDF-15 (GDF-15, MIC-1, MIC1, NAG-1, PDF, PLAB, PTGF-B) Hs00171132_m1 (context sequence: CGCCAGAAGTGCGGCTGGGATCCGG) (Thermo Fisher Scientific).

Amplification was performed using Light cycler 480 systems (Roche Lifesciences). The expression levels of analyzed genes were normalized according to the 18srRNA.

### Cytokine Secretion Assay

The concentration of secreted GDF-15 was determined in macrophage culture supernatants using ELISA assays from R&D systems (Wiesbaden, Germany) according to the manufacturer’s instructions.

### Endocytosis Assay

acLDL-Alexa488 (Invitrogen) was used as a ligand for endocytosis quantification. Endocytosis assays were performed in M0, M1 and M2, in general as described previously (22). Briefly, on day 6, acLDL-Alexa488 was added at a final concentration 2 µg/mL for flow cytometry quantification. For immunofluorescence analysis, macrophages were grown on coverslips, and acLDL-Alexa488 was added at a final concentration of 5 µg/mL. Macrophages were incubated with the ligand for 30 minutes in the presence of 7,5% CO2 at 37°C. Cessation of endocytosis was achieved by placing cells on ice for flow cytometry. For immunofluorescence staining, cessation was achieved by immediate fixation with PFA as described (21), for M0, M1 and M2 treated with TiNPs, and for M1 non-treated with TiNPs. Since M0 and M2 without TiNPs were almost completely suspensional, sample preparation was performed using a Cytospin™ 4 centrifuge (Thermo Fisher Scientific), and cytospins were fixed by PFA.

### Flow Cytometry

Flow cytometry was used to quantify the uptake of the acLDL-Alexa488. After endocytosis cessation, cells were harvested in ice-cold PBS. Fluorescent signal was quantified using BD FACS Canto II flow cytometer (FlowCore Mannheim, Germany). The data were analyzed using FlowJo 10.01 software.

### Immunofluorescence and Confocal Microscopy

The following primary antibodies were used: anti-hstabilin-1 rabbit polyclonal serum RS1 (23) and mouse monoclonal anti–EEA-1 (BD Biosciences). Secondary antibodies were Cy3-conjugated donkey anti–rabbit IgG and Alexa647-conjugated donkey anti–mouse IgG (Dianova, Germany). In addition, all samples were stained with DAPI (Roche, Mannheim, Germany). Specificity of used antibodies was assessed in TiNPs treated cells with appropriate isotype control. Samples were mounted using DakoCytomation Fluorescent Mounting Medium (DakoCytomation, Hamburg, Germany). Confocal microscopy was performed using a Leica laser scanning spectral confocal microscope, model DM IRE2, equipped with an HCX PL Apo 63 ×/1.32 numeric aperture oil objective (Leica Microsystems, Wetzlar, Germany). Excitation was done with an argon laser emitting at 488 nm, a krypton laser emitting at 568 nm, and a helium/neon laser emitting at 633 nm. Images were acquired using a TCS SP8 DLS Leica inverted microscope and Leica Confocal software, version 2.5 (both from Leica Microsystems). Images were acquired using a sequential scan mode. For panel assembly, Adobe Photoshop version 6.0 (Adobe Systems, San Jose, CA) was used. Quantification of the fluorescence intensity for stabilin-1 and acLDL-Alexa488 was assessed using Qupath open source program. Three independent donors were analyzed and five different fields per donor were used for quantification. The program recognized individual cells and quantified the intensity of the signal for each cell. The means of fluorescent intensity were calculated for each field, for each donor, and, finally, for each condition: with or without TiNPs. The average fluorescent intensity of the overall cells was calculated.

### Statistics

Statistical analysis was performed using GraphPad Prism 8 software (GraphPad Software Inc., USA). Bar graphs show mean ± SEM. The significance of the data was analyzed using ratio paired Student’s t-test. We considered a two-tailed p-value of less than 0.05 to indicate statistical significance (confidence level 95%). ns = non-significant, p < 0.05, p ≤ 0.05**, p ≤ 0.01, ***p ≤ 0.001 and ****p ≤ 0.001.

## Results

### Microarray Analysis

**Figure 1:**
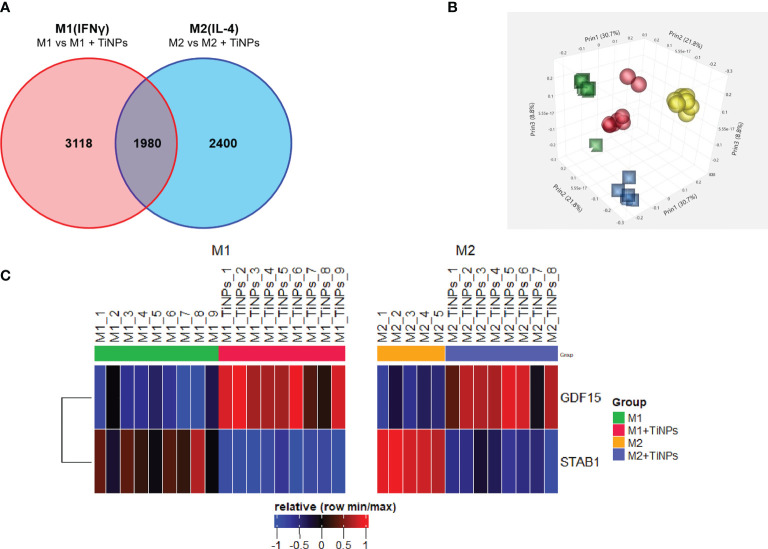
Summary of microarray analysis of the effect of titanium nanoparticles on transcriptome of human inflammatory and healing macrophages. **(A)** Venn’s diagram of differentially expressed genes in macrophages cultured in presence of TiNPs. Each number represents the number of genes differentially expressed in response to TiNPs. Each circle represents a population of macrophages with a different stimulation: Red – M1(IFNγ) and Blue – M2(IL-4). Intersecting points represent the number of genes differentially regulated by TiNPs in both stimulations conditions. **(B)** Clustering of the microarray data in a 3D scatterplot. Each sphere represents genes from one donor. Clustering of data from M1(IFNγ) is represented by yellow spheres – Control and red spheres – TiNPs); clustering of data from M2(IL-4) is represented by blue squares – Control and green squares - TiNPs). **(C)** Heatmap of relative expression of stabilin-1 and GDF-15. Differential expression is shown between Control and TiNPs conditions M1 and M2, respectively. The number of donors differs for every condition depending on the availability of materials.

Microarray analysis revealed that TiNPs at a concentration of 100ppm altered the expression of 5098 genes in M1 (IFN-γ-stimulated) and 4380 genes in M2 (IL-4–stimulated) macrophages. Additionally, 1980 genes were differentially regulated in both M1 and M2. The analysis revealed a significant downregulation of the multifunctional scavenger receptor stabilin-1 under TiNPs in M1 and M2 (fold change: -3,12 and -3,93, correspondingly). A contrary effect was seen for GDF-15, which was transcriptionally upregulated with TiNPs in both activation states (fold change: 2,94 and 2,54, correspondingly). **Figure 1** shows the microarray analysis, in which every comparison was made between cells cultured with TiNPs versus cells without. The original array data for all differentially activated genes is available on the NCBI Gene Expression Omnibus (GEO) browser (Reference number GSE179543).

### TiNPs Stimulate GDF-15 and Suppress Stabilin-1 Gene Expression During Monocyte/Macrophage Differentiation

**Figure 2:**
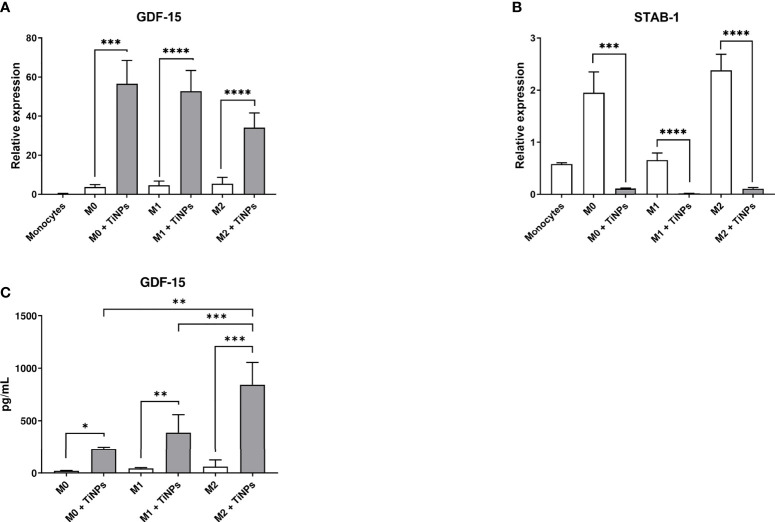
Titanium nanoparticles have opposite effect on the expression of GDF-15 and stabilin-1 and strongly stimulate GDF-15 secretion in macrophages. mRNA levels were analyzed by RT-PCR in monocytes (day 0), and in non-stimulated (M0), M1 and M2 macrophages cultured for 6 days. **(A)** GDF-15 expression: n = 3 for monocytes, n = 7 for M0, n = 12 for M1, and n = 12 for M2. **(B)** Stabilin-1 expression: n = 4 for monocytes, n = 7 for M0, n = 12 for M1, and n = 12 for M2. **(C)** Supernatants of M0, M1 and M2 macrophages cultured for 6 days were analyzed by ELISA: n = 5 for M0, n = 10 for M1, n = 10 for M2. Error bars indicate the means standard error of the results normalized to 18SrRNA expression levels. *p < 0,05, **p < 0,01, ***p < 0,001, ****p < 0,0001.

To confirm the results of the microarray analysis, the expression of GDF-15 and stabilin-1 was verified by RT-PCR. The effect of TiNPs exposure on macrophages was examined after 6 days of monocyte differentiation into non-stimulated (M0), M1 and M2 macrophages. The expression of GDF-15 in monocytes (day 0) was minimal (**Figure 2A**). In all macrophage subtypes, the expression of GDF-15 was significantly increased when treated with TiNPs (**Figure 2A**). TiNPs upregulated the expression of GDF-15 in M0 by the average of 15 times (p=0,0002) and in M1 by 10 times (p<0,0001). Likewise, stimulation of M2 with TiNPs resulted in upregulation of GDF-15 expression by 6 times (p<0,0001). Additionally, GDF-15 expression was not significantly different between the different macrophage phenotypes, both with and without TiNPs. These results indicate that the exposure to TiNPs clearly has a stimulatory effect on the expression of GDF-15 in primary human monocyte-derived macrophages, and this expression is not influenced by macrophage differential activation.

In contrast, stabilin-1 expression was inhibited by TiNPs in all macrophage phenotypes (M0 p=0,0008, M1 p<0,0001, and M2 p<0,0001). In monocytes (day 0) and M1, stabilin-1 mRNA levels were lower compared to M0 and M2 (**Figure 2B**).

### TiNPs Promote GDF-15 Secretion in Activated Macrophages

The effect of TiNPs on GDF-15 secretion levels was assessed by ELISA in supernatants collected from macrophages cultured for 6 days (**Figure 2C**). In all macrophage subtypes, the secretion of GDF-15 was tremendously elevated after exposure to TiNPs. Upon the treatment with TiNPs, M0 increased GDF-15 secretion from almost 15 to approximately 380 pg/mL (p=0,0105). M1 also showed increased GDF-15 secretion under TiNPs stimulation (p=0,0011). The highest GDF-15 secretion was observed in M2 under TiNPs stimulation (M0 vs M2 p=0,0086 and M1 vs M2 p=0,0095).

### TiNPs Decrease the Efficiency of Endocytosis

**Figure 3:**
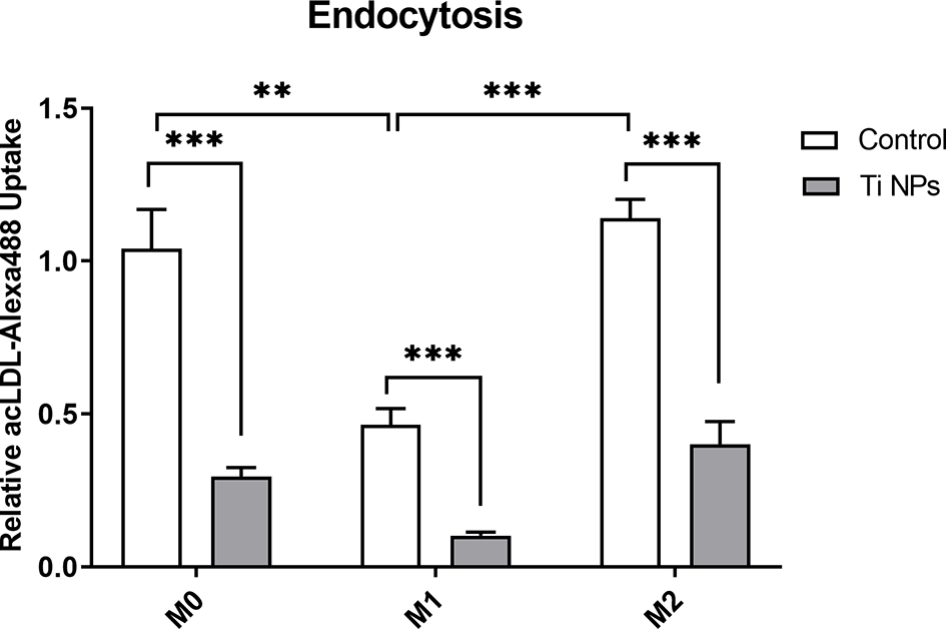
Titanium nanoparticles significantly suppress scavenger-receptor-mediated endocytosis in all types of macrophages. M0, M1 and M2 macrophages were cultured for 6 days. acLDL-Alexa488 was added at a concentration of 2 µg/mL to macrophages for 30 minutes at 37°C. Ligand internalization was quantified by flow cytometry: n = 7. Error bars indicate the means standard error of the geometric mean normalized to non-stained cells relative endocytosis **p < 0.01, ***p < 0.001.

We evaluated the effect of TiNPs on the uptake of fluorescently labeled acLDL, a known ligand of stabilin-1, in M0, M1 and M2 by flow cytometry. As depicted on the graph, the treatment with TiNPs decreased the efficiency of acLDL-Alexa488 endocytosis in all macrophage subtypes (**Figure 3**). After quantification, we found a significantly reduced endocytosis upon exposure to TiNPs compared to controls, which was consistent in all 7 analyzed individual donors. acLDL endocytosis was higher in M0 (p=0,0033) and M2 (p=0,0004) controls as compared to M1 control (**Figure 3**). This data indicates that acLDL endocytosis is negatively affected by TiNPs in all macrophage phenotypes.

### TiNPs Disrupt Stabilin-1-Mediated Endocytic Trafficking

**Figure 4:**
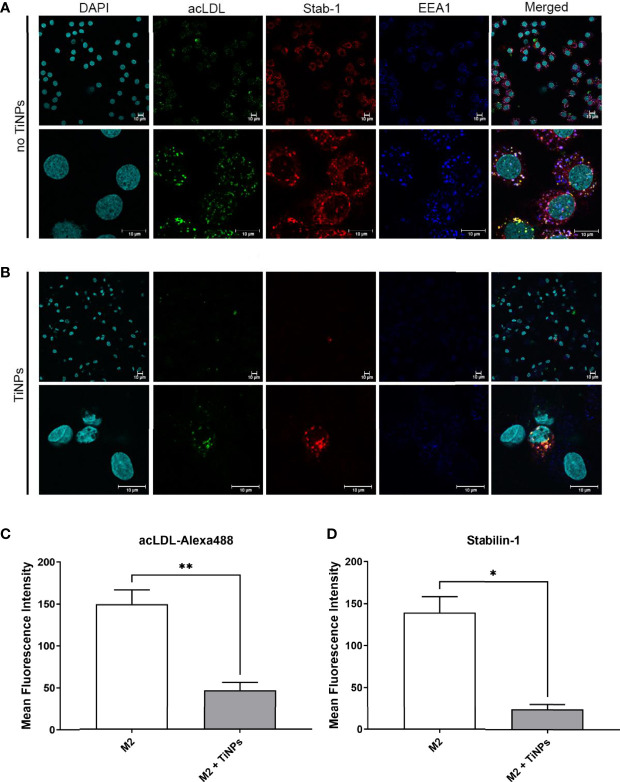
Confocal microscopy analysis of the suppressive TiNP effect on the stabilin-1-mediated endocytosis in healing M2 macrophages. M2 macrophages were exposed to stabilin-1 ligand acLDL-Alexa488 at a concentration of 5 µg/mL for 30 minutes. M2 derived from monocytes of 3 individual donors were used for each type of analysis. **(A)** Representative images of confocal microscopy analysis of M2 in absence of TiNPs. **(B)** Representative images of confocal microscopy analysis of M2 in presence of TiNPs. Visualization of nuclei was performed using DAPI (visualized in cyan). acLDL-Alexa488 is shown in green. Stabilin-1 was detected by RS1 rabbit polyclonal antibody and Cy3-conjugated anti-rabbit secondary antibody (visualized in red). EEA1 was detected using anti-EEA1 mouse antibody and Alexa-647-conjugated secondary antibody (visualized in blue). Representative images of single cells are shown for M2. Scale bars: 10 µm. **(C)** Quantification of confocal microscopy images for the internalized acLDL-Alexa488 and **(D)** quantification of confocal microscopy images for expression of stabilin-1. For every 3 individual donors, 5 different fields of images were assessed using Qupath open source program. The mean fluorescence intensity was calculated by averaging the intensity of each cell per condition. Error bars indicate the means standard error of the results. *p < 0.05, **p < 0.01.

To further explore the effect of TiNPs on stabilin-1-mediated internalization and intracellular transport along the endocytic pathway, confocal microscopy was used to visualize the localization of stabilin-1 and acLDL-Alexa488. Since M2 expresses maximal levels of stabilin-1 and have enhanced endocytic properties compared to M0 and M1, M2 subtype was used for the immunofluorescence analysis (24). EEA1-positive early/sorting endosomes is a major vesicular compartment, where stabilin-1 is localized in M2 (21). Therefore, we assessed its intracellular localization using anti-EEA1 and anti-stabilin-1 rabbit polyclonal RS1 antibody. In the absence of TiNPs, M2 expressed high levels of stabilin-1. Additionally, the majority of acLDL-Alexa488 was co-localized with stabilin-1, and was efficiently delivered to EEA1-positive early/sorting endosomes, while EEA1 marked irregularly shaped large endosomes (**Figure 4A**). Treatment with TiNPs resulted in the disruption of endosomal compartment. Only a small amount of remaining EEA1 endosomes was detected, and internalization of acLDL-Alexa488 was almost abrogated (**Figure 4B**). After TiNPs treatment, stabilin-1 was expressed only in a small percentage of M2, and only in stabilin-1+cells internalization of acLDL-Alexa488 was still detectable. Moreover, the mean fluorescence intensity of both acLDL-488 and stabilin-1 was significantly lower in M2 treated with TiNPs (p=0,008 and p=0,0118, respectively) (**Figures 4C, D**). Overall, the immunofluorescence analysis confirmed the suppressive effect of TiNPs on stabilin-1 expression on protein level and stabilin-1-mediated endocytosis.

## Discussion

Here, the effect of TiNPs (debris of titanium implants) on transcriptome of human healing macrophages was analyzed for the first time. We found that TiNPs suppress essential for healing scavenging function of macrophages, mediated by stabilin-1. At the same time, we found that TiNPs strongly upregulate the production of GDF-15.

GDF-15 is a multifunctional cytokine and remote member from the glial cell-derived neurotrophic factor (GDNF) family and TGF-β superfamily (25, 26). Its membrane receptor, GDNF family receptor α-like (GFRAL), was considered to be uniquely expressed in the hindbrain (25, 27, 28). Recent data showed that GFRAL expression is also present in human adipocytes and prostate cancer cells (29, 30). GFRAL was also reported to be expressed in endothelial cells and to mediate GDF-15-induced pro-angiogenic effect (31).

GDF-15 has been recognized as a potential diagnostic and prognostic biomarker for several diseases, including cancer, cardiovascular disease, sepsis, and recently, COVID-19 (32, 33). GDF-15 is known for being a pleiotropic cytokine mainly expressed and secreted during stress conditions and inflammation, and its function seems to be protective (34–37). Recently, Cimino et al. discovered that GDF-15 induces a rise in glucocorticoid levels through GFRAL signaling upon endoplasmic reticulum (ER) stress induction in mice, which undercovers GDF-15 centrally mediated-anti-inflammatory response (38). Its role in immune tolerance was also addressed by Luan et al, who discovered that GDF-15 rise during bacterial inflammation increases hepatic triglyceride production, mediating cardiac protection and promoting survival (36). Other studies have shown detrimental consequences of increased GDF-15, highlighting a context-dependent action (39). GDF-15 has been correlated with fibrotic diseases. For instance, Govaere et al. quantified GDF-15 expression and secretion levels in hepatic tissue using transcriptomic and proteomic analysis and found that GDF-15 positively correlates to fibrosis progression in nonalcoholic fatty liver disease (NAFLD) (40). Nevertheless, its complete molecular mechanism and signaling pathway have not been fully established.

In macrophages, GDF-15 expression is increased under the effect of IL-4, IL-1β, TNF-α, IL-2 and M-CSF (41–43). Previous studies have found elevated GDF-15 expression in M1 (but not M2) macrophages and alveolar macrophages upon acute exposure to TiNPs and silica microspheres (4 hours and 16 hours, respectively) (19, 44). Our findings complement this information by pointing out a persistent effect of TiNPs (6 days) on GDF-15 gene expression in all macrophage phenotypes. Furthermore, GDF-15 was preferentially secreted by M2 macrophages under TiNPs treatment.

It is worth mentioning that the TiNPs-induced pro-inflammatory reaction has been associated with increased production of reactive oxygen species (ROS) and mitochondrial damage (45). Interestingly, GDF-15 production is induced by mitochondrial uncoupling and by the treatment with mitochondrial inhibitors, including metformin (46, 47). Likewise, TiNPs induce ER stress and disrupt the mitochondrial-associated ER membranes (48). Therefore, identified by us increased GDF-15 expression could be explained by the mitochondrial damage generated by TiNPs. Another documented effect of TiNPs on macrophages is lysosomal damage. Particularly, the here used rutile TiNPs, have a detrimental impact on lysosomal permeability (49). Recently, Kim et al. found that transcription factor EB (TFEB), a regulator of energy expenditure and autophagy inductor, binds to GDF-15 promotor and induced its expression after lysosomal stress induction in macrophages (50). The potential lysosomal damage caused by exposure to TiNPs could also explain the rise in GDF-15 levels. This hypothesis is consistent with the observation from our transcriptome analysis, showing a significant upregulation of TFEB expression (M1: fold change=0,43, p-value=0,03; M2: fold change=1,55, p-value<0,0001) in both human CD14^+^ derived M1 and M2 after 6 days of TiNPs exposure in culture (Reference number GSE179543).

Stabilin-1 is an established biomarker in M2 macrophages essential for their clearance function in health and pathology (51). Multifunctional scavenger receptor stabilin-1 (STAB-1, FEEL-1, CLEVER-1, KIAA0246) is a transmembrane scavenger and sorting receptor expressed on tissue macrophages and non-continuous endothelial cells (21, 24). In human monocyte-derived macrophages, the expression of stabilin-1 is induced by stimulation with IL-4 and dexamethasone (23). Stabilin-1 controls the balance between inflammation and tissue remodeling by scavenging and targeting for secretion multiple extracellular regulatory proteins including SPARC, SI-CLP, YKL-39 and placental lactogen (22, 52–54). It also mediates the clearance of apoptotic bodies as well as modified lipoproteins (55, 56). Here, we found that stabilin-1 expression is highly suppressed under TiNPs treatment in all activation states. Stabilin-1 decreased expression supports the previous observations that TiNPs promote a shift to M1 polarization and suppressed M2 response (19). Our flow cytometry data showed a markedly decreased endocytosis efficiency in all activation states after co-culturing with TiNPs. The fold change was higher for M0 and M2, which are reported to mediate more actively endocytosis than M1 (57). This is also consistent with our observation that M0 and M2 expressed more stabilin-1 than M1. Quantitative confocal microscopy analysis further confirmed the abrogation of stabilin-1 expression in M2 co-cultured with TiNPs. A decrease in stabilin-1 can also result in impaired clearance of SPARC, modified lipoproteins and apoptotic bodies, the accumulation of which is frequent in chronic inflammation and fibrosis (53, 55, 56, 58). Moreover, deficiency of stabilin-1 in mice aggravates inflammation by increased inflammatory macrophage activation and enhanced IgM production (59).

We have previously identified that GDF-15 is an endocytic ligand of stabilin-1 (60). We showed that impaired clearance of GDF-15 in STAB-1–/–STAB-2–/– mice leads to severe glomerular fibrosis and mild perisinusoidal hepatic fibrosis. With this context, the potential implications of increased secretion of GDF-15 added to dysfunction of its clearance receptor in implant holders are numerous. The remark that all macrophage phenotypes highly secreted GDF-15 under TiNPs, highlights the possibility that GDF-15 acts in an autocrine and paracrine matter. For instance, Jung et al. showed that rGDF-15 treatment decreases the expression of IL-6, nitric oxide synthase 2 (NOS2) and TNF-α and promotes an M2 polarization by augmenting Arg-1, Retnla and chitinase 3-like 3 protein (Ym1) production in murine blood marrow-derived macrophages (42). Therefore, GDF-15 could promote a change of phenotype from M1 to M2 in resident and recruited macrophages. Possible receptors for this action are TGF-β RI and II, which were identified to bind to GDF-15 on dendritic cells promoting immune tolerance (61). An accumulation of GDF-15 in the peri-implant tissues could also impact other surrounding cells. Because of its pro-angiogenic properties, increased GDF-15 in the tissue could promote microvasculature proliferation *via* GFRAL (31). Other possible target cells of macrophage-produced GDF-15 are osteoblasts and osteoclasts precursors. Wakchoure et al, for instance, observed that GDF-15 promotes osteolytic lesions in mice models of prostate cancer with bone metastasis (62). Hinoi et al. showed that anti-GDF-15 decreases bone loss and inhibits osteoclastogenesis in mice models (63). Westhrin et al. showed that GDF-15 promotes osteoclast activation in osteoclasts derived from human peripheral blood mononuclear cells while decreasing osteoblast differentiation (64). Contrastingly, Vanhara et al, showed that GDF-15 inhibited the formation of mature osteoclasts in RAW264.7 cells (65). Interestingly, GDF-15 secretion varies with titanium implant surface modifications, being particularly promoted by rough surfaces (66, 67). However, the real impact of GDF-15 on osteogenesis and implant osseointegration is still to be clarified.

Titanium debris have been shown to induce M1-polarization and to significantly increase the production of pro-inflammatory cytokines. IL-1β, IL-6 and TNF-α, which expression and secretion is upregulated in macrophages after exposure to titanium debris, also seem to affect the degree of bone resorption in paracrine matter. Eger et al. demonstrated that the blockade of IL-1β, IL-6 and TNF-α, using neutralizing antibodies, prevents osteolysis due to titanium particles in mouse models (68).These findings highlights the importance of macrophage system on the implant success and opens the door to explore new targetable mechanisms to prevent implant failure, such as stabilin-1/GDF-15.

Titanium implants located in other organs, such as in the heart, can also promote fibrotic reactions *via* increased GDF-15 and decreased stabilin-1 expression. Indeed, GDF-15 has been previously associated with heart remodeling and heart failure (69). Implants containing titanium are frequently used in cardiology, for instance, in patients with reduced ventricular function (70). A release of TiNPs and, consequently, an uncontrolled increase in GDF-15 could in long term promote cardiac fibrosis and further exacerbate heart failure.

Myelocytic depletion after clodronate treatment significantly reduces serum GDF-15 in mice, highlighting macrophages as a significant source of systemic GDF-15 (50). Therefore, it is feasible that macrophage secreted GDF-15 translates into a significant increase of GDF-15 circulating levels, increasing glucocorticoid levels due to TiNPs-induced ER stress, which could systemically lessen the pro-inflammatory effect of TiNPs (38, 48).

In summary, the dual effect of TiNPs on the GDF-15 over-production and strong suppression of stabilin-1 clearance function in macrophages can be a detrimental effect of titanium implant debris formed with the time, which interferes with the long-term healthy implant integration, damages the balance between inflammation and healing processes, and is detrimental for patients. GDF-15 can potentially have also systemic effects and can be considered for therapeutic targeting to eliminate titanium implant-induced complications.

## Data Availability Statement

The datasets presented in this study can be found in online repositories. The names of the repository/repositories and accession number(s) can be found below: https://www.ncbi.nlm.nih.gov/, GSE179543.

## Author Contributions

JK, LS-B, and TS designed the project. LS-B, TS, LS, CT, and CS performed experiments. LS-B, TS, LS, CT, and JK have analysed data. LS-B, JK, TS, and HK wrote the manuscript. All authors contributed to the article and approved the submitted version.

## Funding

The work was performed with the financial support of ERA-NET RUS Plus CoatDegraBac project (for JK).

## Conflict of Interest

The authors declare that the research was conducted in the absence of any commercial or financial relationships that could be construed as a potential conflict of interest.

## Publisher’s Note

All claims expressed in this article are solely those of the authors and do not necessarily represent those of their affiliated organizations, or those of the publisher, the editors and the reviewers. Any product that may be evaluated in this article, or claim that may be made by its manufacturer, is not guaranteed or endorsed by the publisher.
